# The Job Demands and Resources Related to COVID-19 in Predicting Emotional Exhaustion and Secondary Traumatic Stress Among Health Professionals in Spain

**DOI:** 10.3389/fpsyg.2021.564036

**Published:** 2021-03-09

**Authors:** Jennifer E. Moreno-Jiménez, Luis Manuel Blanco-Donoso, Mario Chico-Fernández, Sylvia Belda Hofheinz, Bernardo Moreno-Jiménez, Eva Garrosa

**Affiliations:** ^1^Departamento de Psicología Biológica y de la Salud, Facultad de Psicología, Universidad Autónoma de Madrid, Madrid, Spain; ^2^University Hospital October 12, Madrid, Spain

**Keywords:** COVID-19 crisis, health professionals, job demands, job resources, challenge, emotional exhaustion, secondary traumatic stress

## Abstract

The current COVID-19 crisis may have an impact on the mental health of professionals working on the frontline, especially healthcare workers due to the increase of occupational psychosocial risks, such as emotional exhaustion and secondary traumatic stress (STS). This study explored job demands and resources during the COVID-19 crisis in predicting emotional exhaustion and STS among health professionals. The present study is a descriptive and correlational cross-sectional design, conducted in different hospitals and health centers in Spain. The sample consisted of 221 health professionals with direct involvement in treating COVID-19. An online survey was created and distributed nationwide from March 20 to April 15 which assessed: sociodemographic and occupational data, fear of contagion, contact with death/suffering, lack of material and human protection resources (MHRP), challenge, emotional exhaustion, and STS. Descriptive findings show high levels of workload, contact with death/suffering, lack of MHPR and challenge, and are moderately high for fear of contagion, emotional exhaustion, and STS. We found an indirect significant effect of lack of MHPR on predicting (1) emotional exhaustion through the workload and (2) on STS through fear of contagion, contact with death/suffering, and workload. To conclude, this study examines the immediate consequences of the crisis on health professionals' well-being in Spain, emphasizing the job demands related to COVID-19 that health professionals are facing, and the resources available in these health contexts. These findings may boost follow-up of this crisis among health professionals to prevent them from long-term consequences.

## Introduction

Until recently the novel disease induced by SARS-CoV-2 (COVID-19) has been spreading worldwide. It has been declared as a Public Health Emergency of International Concern (PHEIC; World Health Organization, 2020a), and the pandemic has caused critical global rates of infection, with 2.97 million people infected and 207,000 deaths (retrieved April 27, World Health Organization, 2020a) since the beginning of the pandemic. Data from August 2020 shows differences in the spread of COVID-19 worldwide, including the following critical rates: 21294845 cases globally; 11420860 cases in the Americas; 3754649 cases in Europe; 3040168 cases in South-East Asia; 1723673 cases in the Eastern Mediterranean; 945165 cases in Africa; and 409589 cases in the Western Pacific (World Health Organization, 2020b). Several findings point to Europe as the epicenter of the virus and highlight Italy, Spain, and France as the countries with the fastest infection rates and negative consequences (Ceylan, 2020). Spain has the second-highest rate of people infected and deaths caused by COVID-19 disease in Europe (220000 and 23521, respectively up to April; and 1510023 and 41688 up to November) (Ministry of Health, 2020), which shows the fast spread of the pandemic.

Health professionals were at risk of suffering from several occupational risks before the pandemic (i.e., burnout and secondary traumatic stress) (Blanco-donoso et al., 2018; Moreno-Jiménez et al., 2019), but in the face of COVID-19 encounter several further occupational hazards that may have an immediate psychological impact on well-being (Brooks et al., 2020; Luceño-Moreno et al., 2020; Zhu et al., 2020). Spain has one of the highest rates of health professionals infected by the disease, reaching 40,961 cases in May (Red Nacional de Vigilancia Epidemiológica, 2020). These high rates indicate the need to pay attention to all health professionals fighting against the disease, as they seem to be exposed to stressors of this pandemic as an exceptional crisis (Benfante et al., 2020). Despite the proliferation of scientific papers on this subject, more research is needed that is focused on the impact of the COVID-19 crisis on different health professionals and health contexts.

Concerning the consequences of this crisis, Burnout and Secondary Traumatic Stress (STS) are two negative outcomes widely studied when it comes to health professionals (Kelly, 2020). Firstly, burnout has been considered as emotional exhaustion, depersonalization in professional-patient- relationships, and a lack of accomplishment, due to the high levels of work-related stress (Embriaco et al., 2007). Emotional exhaustion has been commonly considered as the core dimension which better predicts burnout in the short-term (Cieslak et al., 2014), and is considered the outcome of feeling extremely fatigued as a consequence of long exposure to physical, cognitive, and emotional strain due to work conditions. Looking closely, recent studies focused on burnout in health professionals during this COVID-19 crisis in Spain have revealed a critical rate of 41% among these health professionals suffering from emotional exhaustion (Luceño-Moreno et al., 2020).

STS has been defined as the stress resulting from helping or wanting to help others who are suffering a traumatized event (Figley, 1999; Morrison and Joy, 2016). Moreover, STS has been explored in those health professionals more secondarily exposed to traumatic events and for example occurs in health professionals such as those working in Intensive Care Units (hereinafter ICU) (Meadors et al., 2010; Van Mol et al., 2015). However, current findings based on COVID-19 studies have established that the pandemic has increased exposure to traumatic stimuli, such as the fear of contagion, fear of infecting relatives, or increasing rates of deaths (Luceño-Moreno et al., 2020). This impacts directly on all health professionals and increases the risk of developing STS, regardless of specialization. Explaining possible risk factors could help to prevent these negative outcomes. The Job Demands-Resources model (JD-R; Bakker and Demerouti, 2017) provides a way of measuring empirical evidence to theoretically explain the development of both occupational hazards, even in this specific COVID-19 outbreak (Sinclair et al., 2020).

The JD-R model established that job demands are directly and positively related to burnout, and particularly to emotional exhaustion (Bakker et al., 2004). Furthermore, current studies also address other job demands presented in the health contexts (i.e. ethical decision making, the contact with death/suffering, the emotional management of patients/relatives, and the time and social pressure for caring tasks) as strongly related to STS (Moreno-Jiménez et al., 2019, 2020). The JD-R model also supports the idea that the presence of either job or personal resources may diminish the burden of job demands (Bakker and Demerouti, 2017). Within the COVID-19 crisis, the need to examine the job demands and resources presented in health contexts is undeniably relevant in preventing the development of negative outcomes such as burnout and STS. Health professionals are on the “battlefront” in fighting against the disease and are exposed mainly to the high job demands presented during this crisis (Jiang et al., 2020), including a lack of both material and human resources, at the time infections rose (Giusti et al., 2020; Lai et al., 2020). For that purpose, we considered all health professionals within hospitals and health centers as affected by this crisis.

### Job Demands of COVID-19 Outbreak in Health Professionals

Based on the JD-R model, job demands are defined as those physical, cognitive, social, or emotional aspects of a job that require an effort to overcome them and involve a cost (Bakker and Demerouti, 2017). Following this definition, the current situation imposed by COVID-19 disease has resulted in health professionals experiencing long exposure to high workloads, which may have a short-term impact on their psychological well-being (Jiang et al., 2020), and which is associated with more emotional exhaustion (Bakker and Demerouti, 2017). Increases in deaths and infection rates mean it is more likely that they will come into contact with death and the suffering linked to the new phenomenon of fear of contagion (Huang et al., 2020) which may increase the risk of developing STS (Cai et al., 2020). For this reason, contact with death/suffering, fear of contagion, and workload were selected as the outstanding job demands-related to COVID-19, affecting all health professionals in different health contexts as the scenario of this crisis.

### Job Resources of COVID-19 in Health Professionals

In response to the increase in workload, new units have been created to attend to patients infected by COVID-19, extending the crisis impact to affect not only the ICU but also related healthcare units. Consequently, this increase in people infected and the massive use of the ICUs are linked to a depletion of resources. It is noteworthy that the lack of both material resources (i.e., personal protection equipment), due to the increased number of people infected, and the human resources, in turn, related to the increase in infected health professionals. The rate of health professionals infected therefore rose by the time the disease was spreading. This challenges the standard capacity of the caring tasks of those professionals, having fewer resources, and a higher workload (Del Rio and Malani, 2020). Based on the JD-R model (Bakker and Demerouti, 2017), job resources are considered as “those aspects of the job that are functional in achieving work goals and reduce job demands and the associated physiological and psychological costs” (Bakker et al., 2004, p. 86). The lack of material and human protection resources in the current crisis may increase these job demands, not only in terms of workload burdens as mentioned before, but also in terms of those tasks related to COVID-19, increasing risk of contagion and their contact with death and suffering (Cai et al., 2020; Ji et al., 2020). This lack of material and human protection resources may impact job demands, making them more of a hindrance rather than a challenge (Bakker and Sanz-Vergel, 2013), and indeed, making the appearance of negative outcomes more likely (i.e. emotional exhaustion and STS) (Schaufeli et al., 2009).

### Personal Resources of Health Professionals in the COVID-19 Outbreak

Following the JD-R model, personal resources are considered as “beliefs people hold regarding how much control they have over their environment” (Bakker and Demerouti, 2017, p. 275). In challenging crises situations, as seems to be the COVID-19 outbreak, the approach to variable challenges faced by those with hardiness personality have been studied to examine protector factors for emotional exhaustion, which boosts the perception of a difficult situation as a way to grow and learn, making stimulating such difficulties (Henderson, 2015; Ladstätter et al., 2018). This challenge, as a personal resource, depicts an adventurous and exploring approach to live events (Bartone and Bowles, 2020), that enhances the perception of difficulties as challenging and in turn, activates resources to overcome them. Thus, interpretation of a crisis as a challenge means people quickly engage and adapt to these situations (Johnsen and Saus, 2019).

Moreover, several findings on how the hardiness personality faces challenges have been revealed to protect against STS in emergency professionals, by giving significant meaning to the traumatic tasks as a way to learn new competencies and as an opportunity for personal growth (Moreno-Jiménez et al., 2008). This vision allows them to gain more experience by the time they are more involved (Johnsen and Saus, 2019). It seems that people with high adaptability challenges can respond to critical stimuli more quickly and effectively, due to their way of seeing the difficult scenario as a learning context. This challenge could be considered relevant in a crisis, especially in more individual stress control in highly stressful jobs to prevent burnout (Bartone and Bowles, 2020) and specifically among health professionals (Maramis and Cong, 2019).

This study aims to explore these job-related demands (i.e., fear of contagion, contact with death/suffering, and workload) and job-related resources (i.e., the lack of material and human protection) specifically during the COVID-19 crisis, as well as a personal resource (i.e., challenge) that may hinder this negative effect on health professionals, based on the well-established JD-R model. The main contribution of this research relies not only on the study of the high prevalence of the negative outcomes due to the COVID-19 outbreak but also provides a theoretical basis that could boost knowledge of this crisis. This study examines the following hypotheses:

*H*_1_ *The job demands (i.e., fear of contagion, contact with death/ suffering, and workload), the job resources (i.e., the lack of material and human protection resources), personal resource (i.e., challenge) and negative outcomes (i.e., burnout and STS) will not have any difference among different units in health contexts and type of professionals in this COVID-19 outbreak*.

*H*_2_ *Fear of contagion, contact with death/suffering, and workload, as job demands in this COVID-19 outbreak, are positively related to (a) emotional exhaustion and (b) STS*.

*H*_3_ *The lack of material and human protection resources, as a job resource in this COVID-19 outbreak, is positively related to (a) emotional exhaustion and (b) STS*.

*H*_4_ *Challenge within hardiness personality, as a personal resource, is negatively related to (a) emotional exhaustion and (b) STS*.

*H*_5_*. The job demands related to COVID-19 (fear of contagion, contact with death/suffering, and workload) mediate between the resources (the lack of material and human protection resources and challenge) and (a) emotional exhaustion and (b) STS*.

The lack of material and human protection resources will be related to high emotional exhaustion and STS. This is possibly due to the increase in job demands provoked by a perception of the lack of resources.

**Figure 1:**
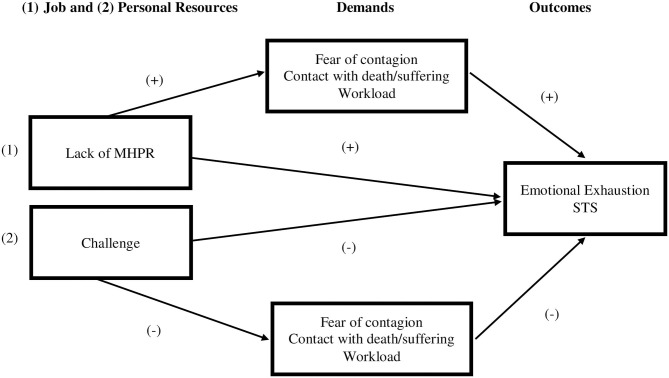
The research model proposed. MHPR, Material and Human Protection Resources; STS, Secondary Traumatic Stress.

Figure 1 represents the model proposed.

## Materials and Methods

### Participants

The study included 221 health professionals from different public hospitals and health centers in Spain, including 78.7% female and 21.3% male participants, with an average age of 40.31 years. The sample was composed of different job positions, the majority of the sample nurses (45.2%) and physicians (33%). Moreover, the sample was classified in different units (depending on where they were working by the assessment time), where the health professionals of ICU and other health specialization within the hospitals (i.e., intern medicine, oncology, pediatric, urology) were more prevalent, 22.2 and 20.4%, respectively. The average years of work experience of the health professionals were 15.79 years. As an outstanding point, 90% of the sample were exposed to patients with COVID-19 symptoms. The sociodemographic and occupational data of the sample are summarized in Tables 1, 2 respectively.

**Table 1:** Sociodemographic data of the total sample.

	**Total health workers**	
	**(*****N*** **=** **221)**	
**Categorical variables**	***n***	**%**
**Gender**		
Male	47	21.3
Female	174	78.7
**Sentimental Relationship**		
With a relationship	175	79.2
Without a relationship	46	20.8
Quantitative variables	*M*	*SD*
Age	40.31	11.59

**Table 2:** Occupational data of the total sample.

	**Total health workers**	
	**(*****N*** **=** **221)**	
**Categorical variables**	***n***	**%**
**Job position**		
Physician Resident medical intern Nurse Nurse aides Emergency technician Ancillary Psychologist Others health professionals^a^ Missing values	73 13 100 22 2 4 1 4 2	33 5.9 45.2 10 0.9 1.8 0.45 1.8 0.9
**Unit**		
ICU Urgency R&S COVID-19 Other specializations within hospitals^b^ HC Others health services^c^	49 29 22 27 45 43 6	22.2 13.1 10 10.9 20.4 19.5 2.71
**First time working in this unit**		
Yes No	51 170	23.1 76.9
**Contact with covid-19 patient**		
Yes No	199 22	90 10
Quantitative variables	*M*	*SD*
Years of experience in the field	15.79	10.97

*R&S, Reanimation and Surgery; HC, Health Centers*.

a *Radiodiagnostic technician and pharmacy technician are included within this category*.

b *Intern medicine, oncology, psychiatry, pneumology, pediatric, traumatology, and urology are included in this category*.

c *Public Health Services and Prevention of Occupational Risks services are included in this category*.

### Procedure

The general procedure was as follows: firstly, we created the questionnaire using the Qualtrics platform (see https://www.qualtrics.com/es/). Within this online questionnaire, the first screen displayed the information related to the study, the main goals, and the informed consent that the participants accepted. The voluntary nature of the study was stated, as well as the possibility to withdraw it at their convenience. They were told that to register their participation, they could send results via email and that they would be contacted with the questionnaire after. Then, once this online questionnaire was created, we obtained a link to access it. This link was sent nationwide by contacting health professionals via email and social networks (such as Facebook, Instagram, Twitter, and LinkedIn). The sample was collected using the well-known snowball techniques for about 3 weeks (from March 20 to April 15). During this period, Spain declared that it was in an “alarm state” and a national lockdown was implemented, except for health professionals who were providing front-line services. All material and human resources in health contexts were mobilized to attend to all patients infected by the COVID-19. These conditions imposed many restrictions that impeded data collection through methods other than via an online questionnaire. This study obtained approval from the Ethical Committee of the Autonomous University of Madrid (CEI-106- 2059).

### Measures

The instruments included present good reliability indexes (see Table 2):

**Sociodemographic data** such as gender, status, job position, years of work experience, unit in which they are working, and contact with COVID-19 patients. They were asked to answer “in this moment” to specifically assess their work status during the COVID-19 outbreak.

**Fear of contagion**. A 3-item scale was designed to assess their fear of both being infected and infecting others with the virus, including relatives (“I have fear of being infected by the virus”). The response category was a Likert scale ranging from 1 (“*nothing*”) to 4 (“*a lot*”).

**Contact with death/suffering**. The 4-item scale related to this variable in the Nursing Burnout Scale (NBS) was included (Moreno-Jiménez et al., 2000). This scale assessed the pain and suffering related to tasks that involved caring for patients before dying (“I feel pain when patients do not receive the visit of their relatives”). The Likert response scale ranged from 1 (“*totally disagree*”) to 4 (“*totally agree*”).

**Workload**. This variable was assessed through the Spanish version of the Secondary Traumatic Stress Scale (STSS; Meda, Moreno-Jiménez et al., 2012). It consisted of 5 items that assessed the amount of work and time pressures required to develop job tasks (“Sometimes we attend to a second notice without enough time to recovery of the previous one”). The Likert-response scale ranged from 1 (“*totally disagree*”) to 4 (“*totally agree*”).

**Lack of material and human protection resources (MHPR)**. This consisted of a 2-item scale designed *ad-hoc* to assess the subjective perception of lack of both protection materials and human resources (“the lack of individual protection equipment scares me” and “the lack of the necessary human resources scares me”). An open section was included at the end of the questionnaire so that participants could add comments finding among these comments a common complaint of lack of material and human protection resources during this crisis. The Likert-response scale ranged from 1 (“*nothing*”) to 4 (“*a lot*”).

**Challenge**. Challenge is a dimension within the hardiness personality, assessed through the Spanish adaptation of Occupational Hardiness Questionnaire (OHQ; Moreno-Jiménez et al., 2014). This variable assesses the natural predisposition to like and feel comfortable in new situations (“At work, I feel more attracted by the innovation and the novelty of procedures”). The scale response ranged from 1 (“*totally disagree*”) to 4 (“*totally agree*”).

**Emotional Exhaustion**. This consists of a 3-item scale included in the Spanish version of the Short Burnout Questionnaire (Moreno-Jiménez et al., 1997). It assesses the physical and mental fatigue related to the caring tasks (“In general, I am rather sick of my job”). The Likert-response scale ranged from 1 (“*nothing*”) to 5 (“*a lot*”).

**Secondary Traumatic Stress**. This outcome was assessed using the Spanish version of the Secondary Traumatic Stress Scale (STSS; Meda et al., 2012). It consists of a 14-item scale in which the cost of being exposed to traumatic events is assessed, in an emotional (“I feel emotionally without strength”) and cognitive (“this work makes me see the world as unfair”) way and the symptomatology related to posttraumatic disorder (“I keep real images about those accidents which affect me a lot”). The Likert-scale response ranged from 1 (“*totally disagree*”) to 4 (“*totally agree*”).

## Results

### Descriptive Analysis

Due to the exploratory nature of this study, means, standard deviations, and Pearson correlations were initially carried out (see Table 3). As observed in Table 3, the job demands related to COVID-19 presented high levels, specifically in contact with death/suffering, and workload and moderately high in fear of contagion. In terms of resources, the lack of MHPR had a high score, as well as a challenge variable. In terms of the outcomes, both emotional exhaustion and STS presented moderately-high levels.

**Table 3:** Means, standard deviations, internal consistency indexes (Cronbach's alpha), and bivariate correlations.

**Variable**	**${\bar{X}}^{a}$**	***SD***	**α**	**1**	**2**	**3**	**4**	**5**	**6**	**7**	**8**
1. Gender	–	–		–							
2. Fear of contagion	2.91	0.75	0.80	0.20^**^	–						
3. Contact with death and suffering	3.58	0.48	0.91	0.37^**^	0.38^**^	–					
4. Workload	3.22	0.56	0.80	0.146^*^	0.34^**^	0.41^**^	–				
5. Lack of MHPR	3.30	0.70	0.68	0.13	0.54^**^	0.30^**^	0.48^**^	–			
6. Challenge	3.05	0.59	0.77	−0.055	0.03	0.11	0.06	0.04	–		
7. Emotional exhaustion	2.75	0.43	0.81	0.77	0.16^*^	0.063	0.30^**^	0.31^**^	−0.26^**^	–	
8. Secondary traumatic stress	2.50	0.88	0.84	0.23^**^	0.39^**^	0.37^**^	0.45^**^	0.43^**^	−0.02	0.60^**^	–

*MHPR, Material and Human Protection Resources*.

Gender was coded as 1= male 2 = female.

All variables are measured from 1 to 4 except for emotional exhaustion which is measured from 1 to 5.

a *1 < 2 = low; 2 < 3 = medium; 3 < 4 = high; 4 < 5 = very high (in case of emotional exhaustion)*.

* p < 0.05;

** *p < 0.01*.

To explore the differences within the sample proposed in H_1_, mean differences through ANOVA were calculated by considering the type of unit (see Table 4) and job position (see Table 5), and Bonferroni statistic was used to make multiple *post hoc* comparison per groups (see note section in Tables 4, 5). Regarding these mean differences, only significant differences were found in lack of MHPR, specifically between health centers and other health specializations (i.e., oncology, psychiatry, pediatric, urology, traumatology, and Public Health Services, see Table 2), being higher in other specializations within the hospital rather than health centers ($\bar{X}=3.56$ and $\bar{X}=3.13$; (95% CI [−0.85, −0.01]; *p* < 0.05). Moreover, significant differences in the challenge were found, specifically between the COVID-19 unit and other health specializations ($\bar{X}=2.77$ and $\bar{X}=3.24$; 95% CI [−0.87, −0.05], *p* < 0.05) and between the latter and health centers ($\bar{X}=2.86$ y $\bar{X}=3.24$, 95% CI [−0.73, −0.01], *p* < 0.05), being higher in other health specializations in both cases (see Table 4). As observed in Table 5, non-significant differences were found regarding job position in none of the interested variables.

**Table 4:** Mean differences between interested units.

	**ICU**	**Urgency**	**R&S**	**Covid-19**	**HC**	**Others^**a**^**	**F**	**Sig**
	***n* = 49**	***n* = 29**	***n* = 22**	***n* = 27**	***n* = 43**	***n* = 51**		
	$\bar{\text{X}}$	$\bar{\text{X}}$	$\bar{\text{X}}$	$\bar{\text{X}}$	$\bar{\text{X}}$	$\bar{\text{X}}$		
Fear of contagion	2.87	2.92	2.85	3.04	2.75	3.06	1.013	0.411
Contact with death/suffering	3.52	3.51	3.47	3.62	3.61	3.70	1.178	0.321
Workload	3.25	3.21	3.36	3.24	2.99	3.31	2.006	0.079
Lack of MHPR	3.18	3.15	3.34	3.44	3.13	3.56	2.762	0.019*^A^
Challenge	3.17	2.97	3.15	2.77	2.86	3.24	3.765	0.003**^B^
Emotional exhaustion	2.58	2.35	2.44	2.60	2.34	2.63	0.799	0.551
Secondary traumatic stress	2.75	2.63	2.69	2.81	2.64	2.90	2.375	0.056

*R&S, Reanimation and Surgery; HC, Health Centers; MHPR, Material and Human Protection Resources*.

*All variables are measured from 1 to 4 except for emotional exhaustion which is measured from 1 to 5*.

a *Other health specialization (i.e., oncology, pneumology, psychiatry, public health)*.

A *Significant mean difference found between health centers and other health specializations (95% CI [-0.85,−0.007]; p < 0.05)*.

B Significant mean differences found between COVID-19 unit and other health specialization (95% CI [-0.87,−0.05], p < 0.05) and between health centers and other health specialization (95% CI [-0.73,−0.009], p < 0.05). *p < 0.05; **p < 0.01.

**Table 5:** Mean differences between job position.

	**Physician^**a**^**	**Nurses**	**Nurse aides**	**Others^**b**^**	***F***	**Sig**
	***n* = 86**	***n* = 100**	***n* = 22**	***n* = 10**		
	$\bar{\text{X}}$	$\bar{\text{X}}$	$\bar{\text{X}}$	$\bar{\text{X}}$		
Fear of contagion	2.81	2.93	3.09	3.16	1.396	0.24
Contact with death/suffering	3.63	3.57	3.51	3.53	0.510	0.67
Workload	3.13	3.28	3.15	3.42	1.799	0.15
Lack of MHPR	3.19	3.38	3.27	3.42	1.237	0.29
Challenge	3.05	2.97	3.23	3.36	2.25	0.08
Emotional exhaustion	2.50	2.55	2.30	2.52	0.477	0.69
Secondary traumatic stress	2.70	2.79	2.82	2.65	1.031	0.38

*MHPR, Material and Human Protection Resources*.

*All response scale was ranging from 1 to 4 except for emotional exhaustion that was from 1 to 5. There were two missing values that did not answer to the job position*.

a *Physician and resident medical intern were taken together*.

b *Due to the small sample, this category is formed by ancillary (n = 4), psychologist (n = 1), emergency technician (n = 2), and other health professionals (n = 4)*.

### Hypothesis Testing

Firstly, to test H_2_ and H_3_, hierarchical multiple regression using stepwise was conducted to establish the possible predictors for both emotional exhaustion and secondary traumatic stress (see **Table 7**). As authors suggest a differential role in terms of gender in the face of COVID-19, gender was included as a control variable (Wenham et al., 2020). The descriptive analysis split by gender is summarized in Table 6. These analyses were conducted using the SPSS 26.0 statistic program.

**Table 6:** Descriptive information per variable concerning gender: males (*n* = 47) and females (*n* = 174).

	**Males**		**Females**	
	**$\bar{\text{X}}$**	**SD**	**$\bar{\text{X}}$**	**SD**
Fear of contagion	2.62	0.69	2.99	0.75
Contact with death/suffering	3.24	0.59	3.68	0.40
Workload	3.06	0.67	3.26	0.52
Lack of MHPR	3.13	0.77	3.35	0.68
Challenge	3.12	0.60	3.04	0.59
Emotional exhaustion	2.47	0.84	2.52	0.90
Secondary traumatic stress	2.56	0.46	2.80	0.42

*MHPR, Material and Human Protection Resources*.

As observed in Table 7, we found a higher explained variance in secondary traumatic stress (*R*^2^ = 0.326; 32.6%), being the greater increment in step 3 with the inclusion of job resources, in this case, the lack of MHPR. In contrast, we found 17,4% of the explained variance in emotional exhaustion, being the greater increment in the last step (step 4) with the inclusion of personal resources, in this case, challenge. (Δ*R*^2^ = 0.072).

**Table 7:** Hierarchical regression model on criterion variables of secondary traumatic stress and emotional exhaustion.

**Predictors**	**Emotional exhaustion**				**Secondary traumatic stress**			
	Standardized β				Standardized β			
Step 1. Control Gender	0.024	−0.002	−0.002	−0.032	0.232^**^	0.091	0.091	0.085
Step 2. Job demands								
Fear of contagionContact with death/sufferingWorkload		0.088 −0.087 0.294^***^	−0.001 −0.085 0.229^**^	−0.005 −0.046 0.234^**^		0.235*^*^ 0.127 0.332^***^	0.147^*^ 0.130 0.268^***^	0.146^*^ 0.138^*^ 0.269^***^
Step 3. Job resourcesLack of MHPR			0.213^**^	0.216^**^			0.200^**^	0.211^**^
Step 4. Personal resourceChallenge				−0.276^***^				−0.056
*R*^2^ Δ*R*^2^	−0.004	0.075 0.071^**^	0.102 0.027^**^	0.174 0.072^***^	0.049	0.299 0.250^***^	0.326 0.027^**^	0.326 0.000

*MHPR, Material & Human Protection Resources*.

*R^2^, Percentage of explained variance by the inclusion of variables*.

* p < 0.05;

** p < 0.01;

*** *p < 0.001*.

In one hand, only workload as job demands seems a positive predictor for emotional exhaustion (*B* = 0.234; *p* < 0.01), as well as the lack of MHPR (β = 0.216; *p* < 0.01). Furthermore, challenge seems a negative predictor for this outcome (β = −0.276; *p* < 0.001). These findings support H_2_ a for workload, along with H_3_a and H_4_a.

On the other hand, all job demands related to COVID-19 seem positive predictors for secondary traumatic stress (see Table 7). Moreover, the lack of MHPR seems a positive predictor as well (β = 0.211; *p* < 0.01), but in this case, we did not find a challenge as a significant predictor so that there is no support for H_4_b based on our results. Thus, our findings support H_2_b and H_3_b.

#### Mediational Effects of Job Demand and Resources

Finally, mediation analysis between the significant resources and job demands related to COVID-19 (fear of contagion, contact to death/suffering, and workload) were carried out. For that purpose, the macro PROCESS was used to calculate the significance of these mediations (see http://processmacro.org/index.html; Hayes and Preacher, 2014). All variables were centered to avoid possible multicollinearity issues.

**Figure 2:**
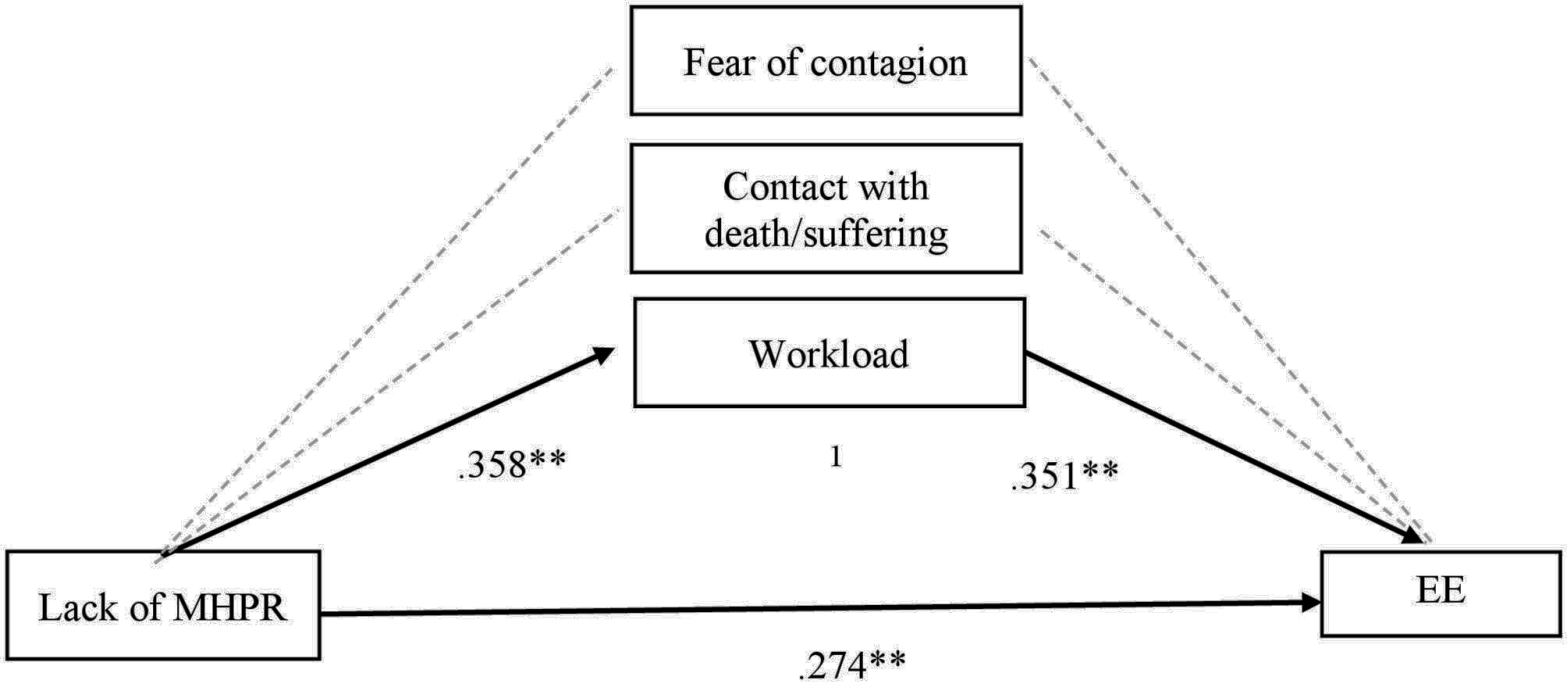
Mediational effects of job demands on lack of MHPR in predicting Emotional Exhaustion (EE). MHPR, Material and Human Protection Resources; EE, Emotional Exhaustion. ^1^Total indirect effect of lack of MHPR on EE through workload (β = 0.12; *t* = 2.97; *p* > 0.01). **p < 0.01.

**Figure 3:**
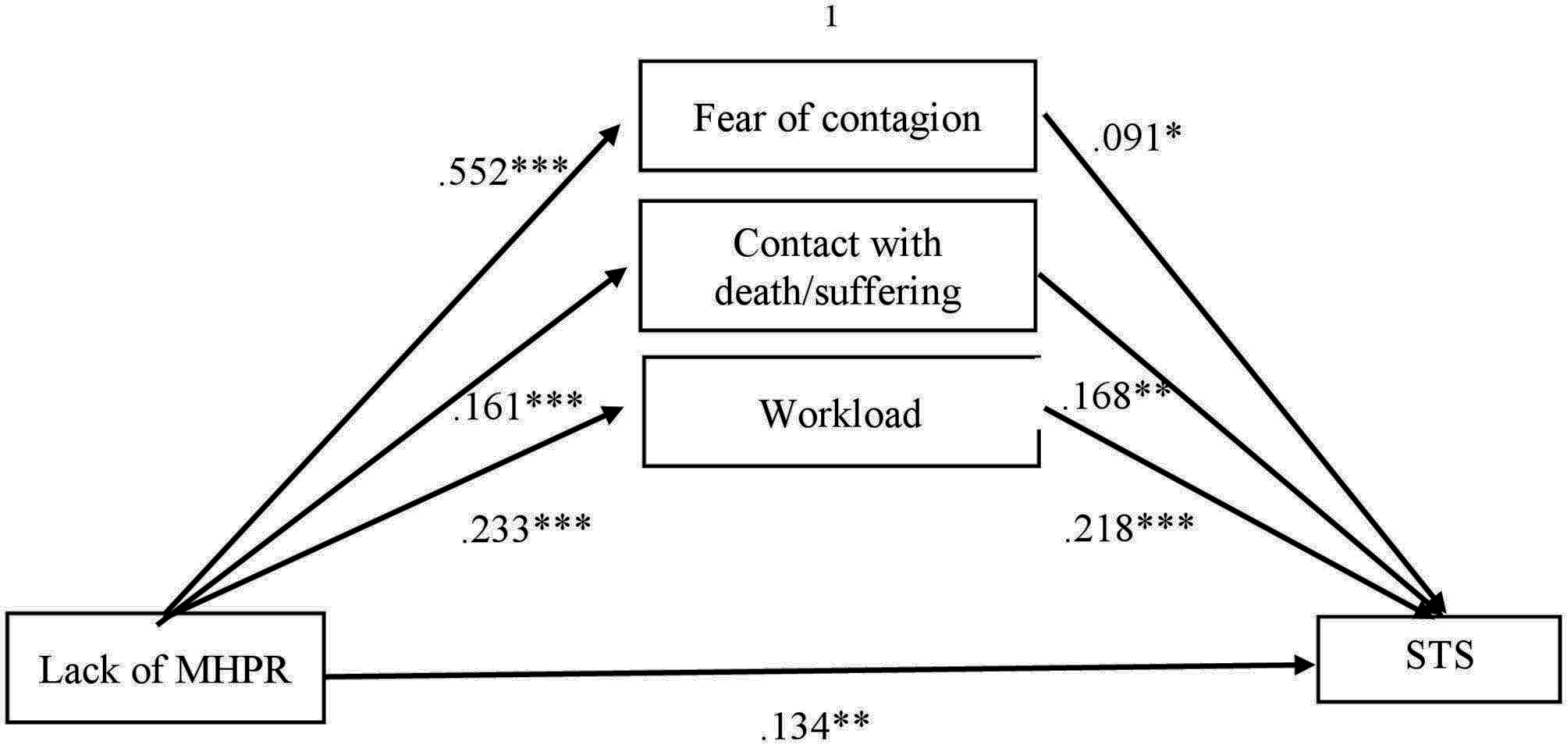
Mediational effects of job demands on lack of MHPR in predicting Secondary Traumatic Stress (STS). MHPR, Material and Human Protection Resources; STS, Secondary Traumatic Stress. ^1^Total indirect effect of lack of MHPR on STS through all job demands (β = 0.139; *t* = 2.976; *p* < 0. 01). *p < 0.05; **p < 0.01; ***p < 0.001.

Due to the lack of a significant relationship between job demands and challenge (see Table 3), we did not find support to test the mediational effect of these job demands between challenge and the outcomes (i.e., emotional exhaustion and secondary traumatic stress), as they need to be significant (Mathieu and Taylor, 2007). Hence, we tested the mediational effect of job demands between the lack of MHPR and both emotional exhaustion and secondary traumatic stress. Figures 2, 3 show these findings.

On the one hand, we found a significant indirect effect of lack of MHPR on emotional exhaustion through workload (see Figure 2). As we observe in this figure, the lack of MHPR positively predicts workload, and this workload leads to more emotional exhaustion (positive predictor). This model explained the 12.8% of the emotional exhaustion variance (*R*^2^ = 0.128), presenting a medium effect size (*E* = 0.09; 95 % CI [.01, 0.10] (Preacher and Kelley, 2011).

On the other hand, we found a significant indirect effect of lack of MHPR on STS through the fear of contagion, contact with death/suffering, and workload. In this sense, observing Figure 3, we can see the lack of MHPR as a positive predictor for fear of contagion, contact with death/suffering, and workload, and these job demands related to COVID-19 lead to more STS (positive predictor). The proposed model explained the 33,7% of the secondary traumatic stress variance (*R*^2^ = 0.337), presenting a medium effect size (*E* = 0.23; 95 % CI [.14, 0.35] (Preacher and Kelley, 2011).

#### Additional Analysis

Prior to contrast mediational effect, we undertook a multiple linear regression to test the possible moderator role of the resources in our model (step 5). We did not find support for the moderator role of either lack of MHPR or challenge between the job demands and emotional exhaustion (*R*^2^ = 0.167; *p* > 0.05) and STS (*R*^2^ = 0.334; *p* > 0.05).

## Discussion

This study aimed to test the impact of the COVID-19 crisis on health professionals working on the front-line of this pandemic by examining perceptions of the job demands (i.e., fear of contagion, contact with death/suffering, and workload) and resources (i.e., lack of MHPR and challenge) during this crisis. Moreover, we aimed to test the effects of a lack of resources in developing negative outcomes, such as emotional exhaustion and secondary traumatic stress. To the best of our knowledge, this is the first study to explore the immediate consequences of the health crisis among health professionals in Spain using a theoretical basis as the JD-R model.

Firstly, our findings provide empirical evidence about the high job demands faced by health professionals among hospitals and health centers. These high levels of job demands are positively predicted by the lack of resources, which highlights the outstanding role of this lack of resources and its relationships with the negative outcomes, as proposed in H_5_. According to previous studies in China, a lack of material and human resources is related to a higher workload, making work shifts more exhausting and even requiring extra work to accomplish all caring tasks required (Jiang et al., 2020). As the authors suggest, working under these stressful conditions and high workloads may increase emotional exhaustion, as these health professionals may not have enough resources to overcome their tasks (Bakker and Demerouti, 2017). This is supported by our findings in H_5_, in which the lack of MHPR is positively related to the workload. This workload alongside emotional exhaustion, have a mediational effect on the workload between this lack of MHPR and emotional exhaustion.

A lack of MHPR, specifically the personal protection equipment during this pandemic, increases the vulnerability of healthcare worlers to contagion and consequently the fear of it (Kang et al., 2020; Lai et al., 2020). This fear of contagion alongside an increase in and near-constant contact with death/suffering, means that health care workers are more likely to develop STS (Kelly, 2020). Spain has one of the highest rates of health professional infection by coronavirus disease, which undeniably highlights the lack of human resources and their fear of contagion which leads to more posttraumatic symptoms (Luceño-Moreno et al., 2020). These conclusions, based on our findings, indicate the mediational effect of workload, fear of contagion, and contact with death/suffering between the lack of MHPR and STS. These findings not only emphasize the positive relationship between high job demands, emotional exhaustion, and STS but also the importance of lack of resources, which may increase job demands and lead to more negative long-term consequences.

Secondly, our findings indicate that the demanding contexts of this crisis faced by health professionals have a similar effect regardless of job position. This result highlights the need to pay special attention to all health professionals working in front-line COVID-19 disease-facing roles. The differences found regarding the lack of material and human resources between the other health specialization within the hospital and the health centers, reveal the extend of the crisis and its qualitative impact. Health professionals of other specializations within hospitals have experienced an increase in job demands and are exposed to the main infection focus (i.e., the increased rate of infected patients within the hospitals), deriving their material resources to these units, mainly focused on infected patients with COVID-19. For this reason, they may experience a lack of resources to a high degree, both material and human, due to the need to allocate health professionals from other specializations to COVID-19-related areas. Linked to that, it is possible that in highly specialized units such as intensive care units, they are most used to working under certain stressors related to workload and time pressure, as well as limited resources (Embriaco et al., 2007). This fact may explain that their perception of the lack of resources could be lower in comparison with other units, although it has worsened during this health crisis.

Remarkably, a positive result may be found regarding challenge as a personal resource. This challenge may protect against the exhaustion derived from the high workload and the lack of material and human resources but seems to not be related specifically to demands related to COVID-19 (i.e., fear of contagion and contact with death and suffering). This is a preliminary result in understanding how personal cognitions and interpretation of the crisis may play a protective role against emotional exhaustion. However, little is known about their interaction in such a demanding context, with higher rates of contact with death and suffering and fear of contagion, meaning these findings require careful interpretation. As an example of this, despite all health professionals presenting a high level of challenge, possibly activated by this crisis, a significant difference appears in other health specializations within hospitals in comparison with other units (i.e., COVID-19 unit and health centers). It could be possible that the qualitative higher levels of contact with death/suffering and type of caring tasks of interacting directly with infected patients on the front line, could change or perturb their perception of challenge, in comparison with those in the second line, as occurs in other health specializations. In this case, challenge profiles could not have a protector effect on STS and have a possible interaction with another hardiness dimension, such as control or commitment, which play a key role in traumatic-related demands (Ladstätter et al., 2018). This fact must be taken carefully as a preliminary result and future longitudinal designs may allow us to temporarily study this along with the crisis and its effect on both outcomes.

The present study remarks on the relevance of taking a closer look into the well-being of health professionals during this crisis. Although the levels of both outcomes are still moderate, the impact of the crisis could be noticed after a period of time, and in this sense, previous studies have pointed out that the psychological impact of this epidemic may last longer than the epidemic itself (Ornell et al., 2020). Because we may have exhausted health professionals, turnover and quit intentions could increase (Moreno-Jiménez et al., 2012), and the quality of care might diminish (Wang et al., 2020). For this reason, currently, studies strongly suggest that the presence of material and human resources is the main motivational factor for health professionals to continue developing their careers (Cai et al., 2020), according to our findings. Furthermore, recent studies about the pandemic in China address the buffering role that leaders may play against the stress burden in this crisis (Jiang et al., 2020).

### Limitations and Future Research

Despite the valuable findings of the present study, some limitations should be emphasized to improve further future research. Firstly, the heterogeneity of the sample aimed to include as many health professionals as possible to gather the real impact of the crisis within all levels. This issue may hinder the applicability of the results, obtaining different levels of exposure and units which may function in different ways (i.e., intensive care units vs. other health specializations within the hospitals). The reason to be inclusive even to health centers was to remark on the extent of the health crisis, and in the second place, to make them part of all preventative measures which should be considered, despite minimizing the effect size of the study. Secondly, the only feasible way to study the real impact in the acute phase of the crisis specifically with the health professionals in the front line was through an online questionnaire using self-report measures. Although this method diminishes ways of objectively assessing the interested variables, obtaining their perceptions and expectations about the job demands and resources during this critical period was crucial to boosting preventative measures from early stages.

Further short and medium-term research should be conducted aiming to surpass these limitations. Based on this, a longitudinal design will be carried out with two goals: (1) to get a follow-up of the impact of the crisis on health professionals' well-being; and (2) to study the different effective coping skills used to overcome this crisis, to be trained after this period, and preventing the participants from a future health crisis, otherwise, we would not learn about the current situation. For these goals, it is important to collect a bigger sample, considering the units and job positions included in this research and establishing better predicting results.

### Practical Implications

Practical implications should be addressed, highlighting the power of prevention and due to the long-term effect that this crisis may have. In the first place, prevention needed a theoretical model to explain the relevant risk factors affecting health professionals. This study provides a valuable theoretical basis using the JD-R model, which allows us to classify the demands and resources to better understand the process. Following our findings, the next steps to prevent long-term negative consequences in health professionals should involve providing greater job resources, from material resources (i.e., personal protection equipment) and staff reinforcements or more co-worker and supervisor support. An increase in job resources could lead to a smaller workload, fear of contagion, and even contact with death/suffering, preventing them specifically from STS and its emotional consequences.

## Conclusions

The current health context involves higher job demands, including not only increased workload, but also traumatic events such as fear of contagion and contact with death and suffering. In this demanding context, addressing this lack of resources is crucial to prevent the development of occupational negative outcomes, such as emotional exhaustion and secondary traumatic stress. This study has revealed that health professionals are facing a critical situation that poses an extra challenge and reflects their motivation toward professions. However, although their challenge allows them to adapt and respond to this crisis, this personal resource should be reinforced by more material and human resources, as well as better working conditions, to diminish the impact of the crisis (i.e., adequate recovery time and less job insecurity, among others). Furthermore, a follow-up of crisis impact should be made to continue caring for those health professionals now on the front line of the crisis.

## Data Availability Statement

The raw data supporting the conclusions of this article will be made available by the authors, without undue reservation.

## Ethics Statement

The studies involving human participants were reviewed and approved by Ethical Committee of Autonomous University of Madrid. The patients/participants provided their written informed consent to participate in this study.

## Author Contributions

The authors presented in this paper have played a role in each phase of the research development. In the first place. JM-J and LB-D with the supervision of BM-J and EG created the online questionnaire and was nationwide send with the collaboration of MC-F and SB. In the second place, JM-J and LB-D were in charge of analyzing the data, being supervised by BM-J and EG. Finally, the first draft of the manuscript was originally written by the first author (JM-J), but being revised, corrected and modified by each author (LB-D, MC-F, SB, BM-J, and EG). All authors contributed to the article and approved the submitted version.

## Conflict of Interest

The authors declare that the research was conducted in the absence of any commercial or financial relationships that could be construed as a potential conflict of interest.
